# Intrinsic Angiogenic Potential and Migration Capacity of Human Mesenchymal Stromal Cells Derived from Menstrual Blood and Bone Marrow

**DOI:** 10.3390/ijms21249563

**Published:** 2020-12-15

**Authors:** Rosana de Almeida Santos, Karina Dutra Asensi, Julia Helena Oliveira de Barros, Rafael Campos Silva de Menezes, Ingrid Rosenburg Cordeiro, José Marques de Brito Neto, Tais Hanae Kasai-Brunswick, Regina Coeli dos Santos Goldenberg

**Affiliations:** 1 Carlos Chagas Filho Institute of Biophysics, Federal University of Rio de Janeiro, Rio de Janeiro 21941902, Brazil; rosana_almeida@ufrj.br (R.d.A.S.); karina_asensi@biof.ufrj.br (K.D.A.); juliahelenadb@gmail.com (J.H.O.d.B.); rafael.camposrj@hotmail.com (R.C.S.d.M.); kasaitais@yahoo.com.br (T.H.K.-B.); 2 Institute of Biomedical Sciences, Federal University of Rio de Janeiro, Rio de Janeiro 21941902, Brazil; ingridrcordeiro@gmail.com (I.R.C.); brito@histo.ufrj.br (J.M.d.B.N.); 3 Department of Life Science and Technology, Tokyo Institute of Technology, Yokohama 2268501, Japan; 4 National Center of Structural Biology and Bioimaging, Federal University of Rio de Janeiro, Rio de Janeiro 21941902, Brazil; 5 National Institute of Science and Technology for Regenerative Medicine-REGENERA, Federal University of Rio de Janeiro, Rio de Janeiro 21941902, Brazil

**Keywords:** menstrual blood-derived mesenchymal stromal cells, bone marrow mesenchymal stromal cells, human umbilical vein endothelial cells, angiogenesis

## Abstract

Several therapies are being developed to increase blood circulation in ischemic tissues. Despite bone marrow-derived mesenchymal stromal cells (bmMSC) are still the most studied, an interesting and less invasive MSC source is the menstrual blood, which has shown great angiogenic capabilities. Therefore, the aim of this study was to evaluate the angiogenic properties of menstrual blood-derived mesenchymal stromal cells (mbMSC) in vitro and in vivo and compared to bmMSC. MSC’s intrinsic angiogenic capacity was assessed by sprouting and migration assays. mbMSC presented higher invasion and longer sprouts in 3D culture. Additionally, both MSC-spheroids showed cells expressing CD31. mbMSC and bmMSC were able to migrate after scratch wound in vitro, nonetheless, only mbMSC demonstrated ability to engraft in the chick embryo, migrating to perivascular, perineural, and chondrogenic regions. In order to study the paracrine effects, mbMSC and bmMSC conditioned mediums were capable of stimulating HUVEC’s tube-like formation and migration. Both cells expressed VEGF-A and FGF2. Meanwhile, PDGF-B was expressed exclusively in mbMSC. Our results indicated that mbMSC and bmMSC presented a promising angiogenic potential. However, mbMSC seems to have additional advantages since it can be obtained by non-invasive procedure and expresses PDGF-B, an important molecule for vascular formation and remodeling.

## 1. Introduction

Over the past few decades, ischemic diseases are the most common causes of deaths in the world. Of this group, ischemic heart disease and stroke are the ones that require great attention [1], currently responsible for approximately 15 million deaths in 2019 [2], but also critical limb ischemia, a serious form of peripheral artery disease, represents the third most prevalent form of atherosclerotic cardiovascular disease [3]. The increasing burden of diseases related to the circulatory system has faced modern medicine to provide therapies that not only mitigate the symptoms of these diseases, but also facilitate the regeneration and function of the affected organs.

The idea of promoting increased blood perfusion in ischemic tissues via angiogenesis may be promising. Considering that ischemic disorders can be primarily caused by endothelial dysfunction, the logic behind therapeutic angiogenesis would be related to the promotion of vascular repair via endothelial cells and/or administration of growth factors [4,5,6,7]. However, the low efficiency of mature endothelial cells expansion as well as the reduced availability of endothelial progenitor cells (EPC) in postnatal tissues, generate limitations for the applicability of these cells in angiogenic therapy, in most cases [6,8]. On the other hand, mesenchymal stem/stromal cells (MSC) are multipotent cells that do not encounter such problems, can be isolated from several adult tissues, have low immunogenicity, and can be easily expanded in vitro in relatively short intervals [9,10,11,12,13,14,15,16,17,18,19]. In addition, several studies pointed out the beneficial effects of MSC in different disease models, mainly due to the paracrine secretion of pro-angiogenic, anti-apoptotic and anti-inflammatory trophic factors that can contribute to tissue repair [20,21,22,23,24,25]. The regenerative potential of MSC is so promising that several groups are already developing delivery methods of MSC on a 3D cell-delivery platform, which can be an alternative to improve cryoprotection, graft survival, angiogenic activity and prolonged cell function in vivo in order to explore the full potential of MSC in regenerative medicine [26].

Among the MSC sources extensively explored and used in the clinic [12,27,28,29,30,31], bone marrow mesenchymal stromal cells (bmMSC) are worth mentioning. However, it is hard to find donors, since human bone marrow is obtained by invasive and painful surgical procedure that use needles to withdraw liquid marrow from both sides of the pelvic bone. In this scenario, the search for other MSC sources was triggered. The discovery of menstrual blood as an MSC source offered a simple, safe, and painless alternative to bypass the invasiveness procedure [32,33]. The MSC obtained from different sources have different safety and efficacy profiles whose complete characterization is a sine qua non condition for their future use in clinical therapies [34]. Similar to MSC from other sources, menstrual blood-derived mesenchymal stromal cells (mbMSC) have great clonogenic capacity and multipotentialities in vitro [33,35,36,37,38,39,40,41]. The release of trophic factors as well as the modulation of the immune response are also beneficial aspects and make the clinical application of mbMSC even more interesting [18,42,43,44,45].

Considering that uterine endometrium undergoes intense ischemia followed by angiogenesis during the menstrual cycle, it is plausible that the MSC present in the endometrial environment and menstrual fluid could present advantageous angiogenic properties in relation to bone marrow MSC. In addition, once their angiogenic potential is confirmed, MSC derived from menstrual blood could be a more viable and renewable source for clinical application in ischemic disease therapies. Since there are few studies in the literature that compare the angiogenic potential of both MSC, this work sought to analyze and compare the angiogenic paracrine effects and intrinsic angiogenic capacity of these cells in vitro, besides to evaluate in vivo the possible contribution to angiogenesis of both MSC during chick embryo development.

## 2. Results

### 2.1. Intrinsic Angiogenic Potential of MSC

**Figure 1:**
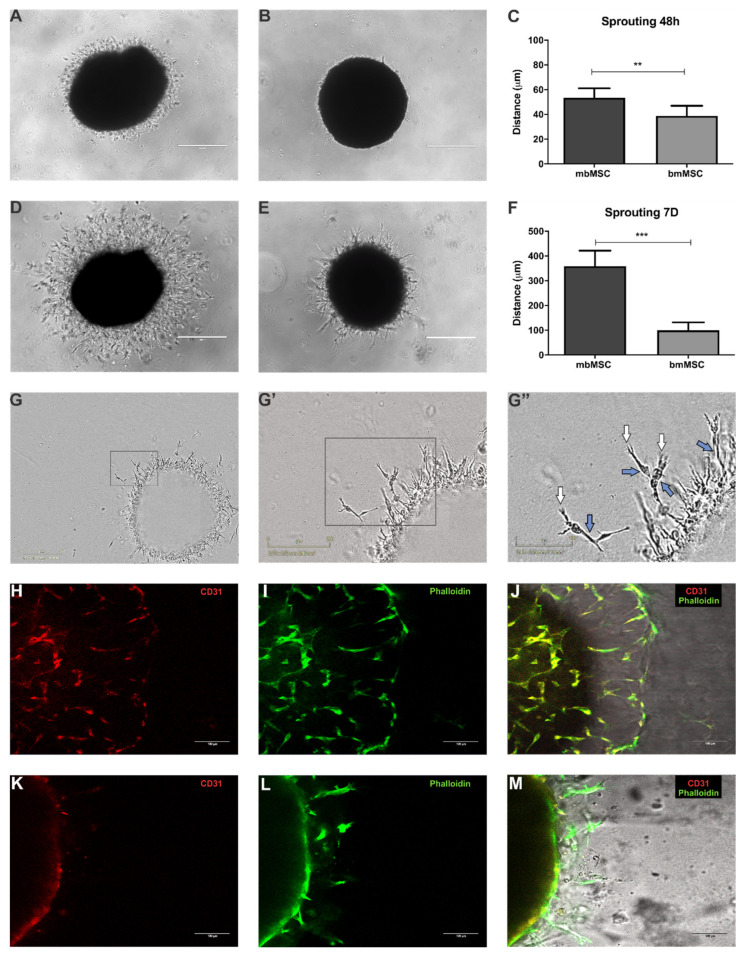
Sprouting from the 3D spheroids formed by mbMSC (*n* = 7) and bmMSC (*n* = 6). Representative images of (**A**) mbMSC and (**B**) bmMSC spheroids after 48 h in Matrigel^TM^ GFR with EGM-2 medium. mbMSC spheroids showed more invasive cells compared to bone marrow spheroids. (**C**) Quantitative analyses of sprout length after 48 h. Distance reached by mbMSC spheroids was significantly longer than bmMSC spheroids (50.77 ± 8.80 µm vs. 38.76 ± 8.19 µm, ** *p* < 0.01). Representative images of (**D**) mbMSC and (**E**) bmMSC spheroids after 7 days in Matrigel^TM^ GFR with EGM-2 medium. mbMSC spheroids showed extensive development while sprouting of bmMSC spheroids was significantly reduced. (**F**) Quantitative analysis of sprout length after 7 days. Distance quantification of mbMSC-derived spheroids was significantly longer than of bmMSC (358.51 ± 62.44 µm vs. 99.99 ± 31.89 µm *** *p* < 0.0001). (**G**–**G”**) Representative images of mbMSC invasive cells in different magnifications showing morphological characteristics similar to endothelial cells. (**G”**) Presence of filopodia-like extensions in their extremities, recapitulating morphologically to tip cells as indicated by white arrow and linear organization similar to stalk cells as indicated by blue arrow. (**H**–**M**) Immunofluorescence of mbMSC (*n* = 6) and bmMSC (*n* = 5) spheroid after 14 days in culture. (**H**–**K**) mbMSC and bmMSC expressed CD31 during angiogenic sprouting assay as shown in red. (**I**,**L**) Cell’s cytoskeleton is shown in green as indicated by phalloidin staining. (**J**,**M**) Merged images of immunofluorescence and phase contrast. All scale bars are indicated in the images.

Sprouting assay evaluated the response of mesenchymal stem/stromal cells in relation to the initial stages of angiogenesis. After 48 h of cultivation in Matrigel^TM^ GFR, many invasive cells appeared at the edge of the spheroid formed by the MSC of menstrual blood, with an average distance of 50.77 ± 8.80 µm (Figure 1A,C). On the other hand, the same was not observed in spheroids formed by bone marrow-derived MSC, which in turn, showed few cells beginning to invade the surrounding matrix and achieved shorter distances (38.76 ± 8.19 µm) (Figure 1B,C). When the culture period was extended to 7 days, spheroids exhibited both single-cell invasion and coordinated, multicellular cord-like structure formation with numerous branches in all directions around the spheroid (Figure 1D,E). However, sprouting development of mbMSC was much more branched and reached longer distances when compared to bmMSC (358.51 ± 62.44 µm for mbMSC verses 99.99 ± 31.89 µm, for bmMSC; Figure 1F).

Additionally, both MSC, in the most apical portion of the invasive cells, acquired morphological characteristics similar to endothelial cells during angiogenic sprouting, as shown by representative images of mbMSC spheroid in Figure 1G–G”. Invasive cells were highly branched, with the presence of filopodia-like extensions in their extremities, being morphologically similar to tip cells (white arrow; Figure 1G”), which are activated endothelial cells that initiate angiogenic sprouting. In addition, MSC of the growing sprout, located just behind the cells of the extremity, were organized in a linear way and also reminds stalk cells (blue arrow, Figure 1G”).

Our next step was to investigate whether menstrual blood and bone marrow-derived MSC could also be differentiated into an endothelial-like phenotype, since morphological characteristics similar to tip cells and stalk cells were observed during the development of angiogenic sprouting. Immunofluorescence performed 14 days after plating sprouting experiment and in the presence of angiogenic endothelial medium revealed that 83.3% of the mbMSC (*n* = 5/6) and 60% of the bmMSC’s spheroids (*n* = 3/5) evaluated expressed the CD31 molecule (Representative Figure 1H,K), suggesting that these cells could also be differentiating into an endothelial-like phenotype. Moreover, the filamentous actin of cytoskeleton was also stained with anti-phalloidin antibody (Figure 1I,L). Merged images of mbMSC and bmMSC are shown in Figure 1J,M, respectively.

### 2.2. In Vitro Migration Capacity of MSC

**Figure 2:**
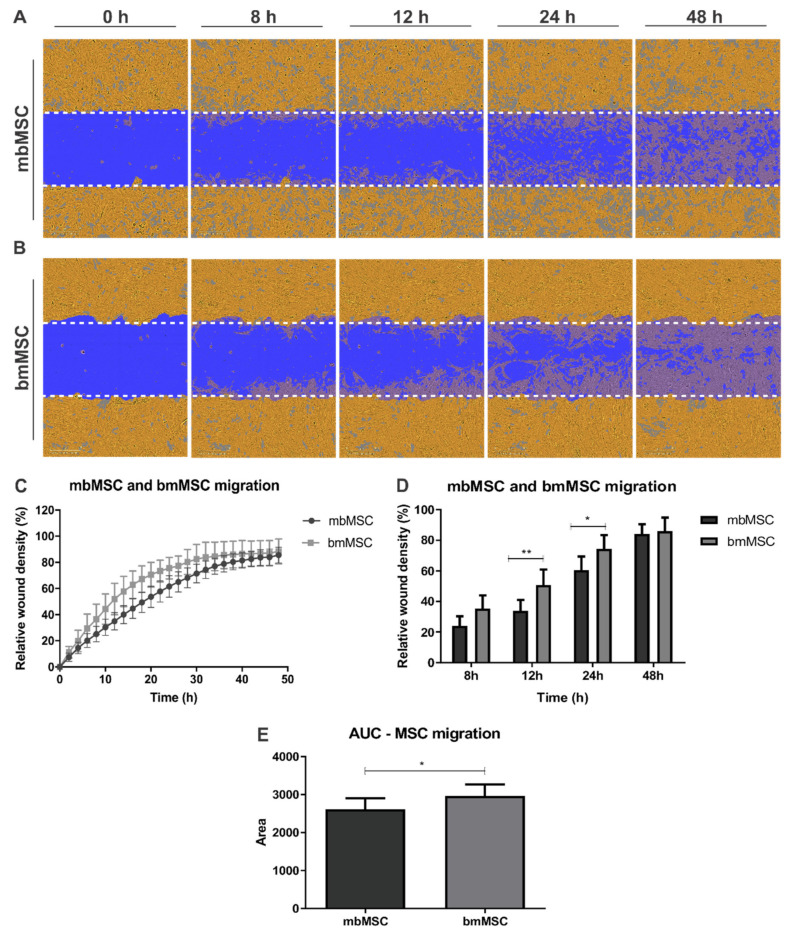
MSC migration capacity in vitro during scratch wound assay. (**A**,**B**) Representative images of (**A**) mbMSC and (**B**) bmMSC migration at the experimental times of 0, 8, 12, 24 and 48 h after scratch was performed. Cells around the wound are represented in orange, wound is represented in blue and migrated cells are represented in gray inside the blue region. Scale bars are 300 µm. (**C**) Migration curves of mbMSC (*n* = 7) and bmMSC (*n* = 7) over the 48-h experimental protocol. (**D**) Relative cell densities quantification at different time points after scratch was performed (mbMSC versus bmMSC after 12 h, ** *p* < 0.01 and mbMSC versus bmMSC after 24 h, * *p* < 0.05). (**E**) Area under the curve quantification indicated that bmMSC migrated faster than mbMSC (* *p* < 0.05).

Scratch wound assay was performed with both MSC to assess their chemotactic motility in response to an injury stimulus. Percentages of relative wound density were quantified in each time point, over 48 h, in order to evaluate scratch closure. Representative images used for this quantification of mbMSC and bmMSC are shown in Figure 2A,B, respectively. In addition, representative videos of mbMSC (Video S1) and bmMSC (Video S2) migration are available on Supplementary Materials. It is worth mentioning that at the end of 48 h, the wound region is practically all occupied by both cell types. Migration curves were constructed for each cell type and revealed a greater slope of the bmMSC curve in relation to mbMSC, mainly in the initial 20 h of the experiment (Figure 2C).

In addition, bmMSC occupied a larger area of the wound region at the experimental times of 12 h (*p* < 0.01) and 24 h (*p* < 0.05) compared to mbMSC. However, at the last time point, in 48 h, densities in the wound region for both cell types become similar (86.24 ± 6.69% for mbMSC and 85.99 ± 8.83% for bmMSC; Figure 2D), indicating that the mesenchymal cells from menstrual blood and bone marrow were able to reach the injury site and possibly exert its regenerative effect. Moreover, the area under the curve was also calculated in order to estimate migration velocity and showed that bmMSC occupied the wound region significantly faster over the experimental period, probably by the greater slope at the beginning (Figure 2E).

### 2.3. In Vivo Migration Capacity and Survival: A Xenograft Assay Using Chick Embryos

The xenograft assay was carried out with the purpose of identifying the location of the studied cells in a highly instructive and dynamic embryonic microenvironment, investigating whether MSC could migrate to specific regions in response to embryonic signals, as well as assessing whether these cells could participate in processes related to vasculogenesis and angiogenesis in vivo. Spheroids, instead of cell suspension, were used to ensure better accuracy and handling at the time of engraftment, as well as to allow assessing whether the cells would migrate from the implanted site to other regions of the embryo.

**Figure 3:**
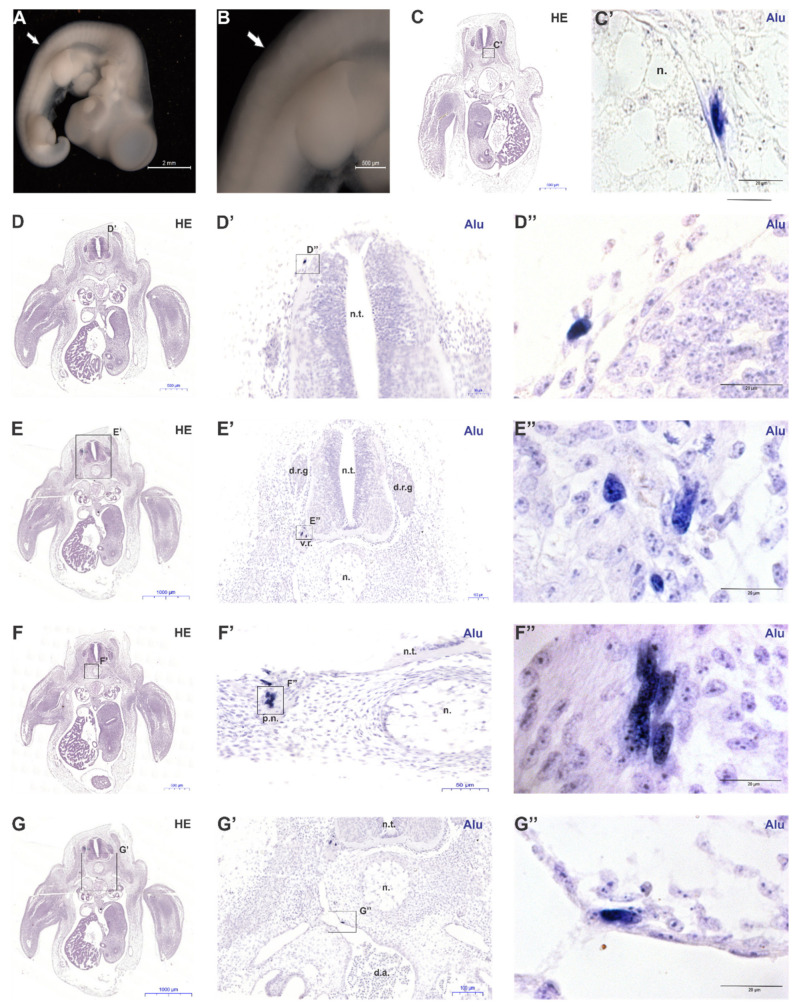
In vivo migration capacity of mbMSC during embryo development. (**A**,**B**) Representative photomicrographs of a E4.5-5 chick embryo after spheroid implantation. White arrows indicate a scar in the somatic region, where the engraftment was performed. (**C**–**G**) Hematoxylin and eosin staining of cross sections at the wing level. (**C’**–**G’**,**C”**–**G”**) Sections adjacent to (**C**–**G**) counterstained with hematoxylin and hybridized with *Alu* probes. (**C’**) Presence of *Alu*-positive mbMSC lateral to the notochord, in presumptive chondrogenic regions. (**D’**–**D”** and **E’**–**E”**) *Alu*-positive mbMSC were close to the basal lamina of the neural tube and ventral root, respectively. (**F’**,**F”**) *Alu*-positive mbMSC were associated with developing peripheral nerves. (**G’**,**G”**) Presence of *Alu*-positive mbMSC in direct contact with the dorsal aorta endothelium of the chick embryo. All scale bars are indicated in the images. N.t., neural tube. d.a., dorsal aorta. n., notochord. d.r.g., dorsal root ganglion. v.r., ventral root. p.n.: peripheral nerve.

Fifty percent of the embryos used for grafting the spheroids were alive (*n* = 4 for each cell type) three days after surgery (E4.5—5, HH27). The engraftment caused a slight scar verified by a mild depression in the region of the cervical somites of the spinal column, as indicated by white arrows in Figure 3A,B. Nevertheless, this depression did not impair embryonic development and morphogenesis after visual and microscopic analyses.

Cross-sections hybridized with *Alu* probes showed that mbMSC migrated along with embryonic cell populations and integrated into host tissues. It is important to note that no cellular aggregate was found in the mesenchyme laterally to the neural tube, suggesting that these cells have mostly migrated to different regions of the chick embryo. *Alu*-positive mbMSC were located close to the notochord, representing 8.3% of total cells found (*n* = 2; Figure 3C,C’). It is worth mentioning that cells derived from the sclerotome of somites migrate to these regions to form the future vertebrae of the axial skeleton of the embryo. Although endochondral ossification has not started at this stage of development, the notochord and surrounding area can be considered presumptive chondrogenic regions. So, mbMSC showed tropism for regions that form cartilage, corroborating to the chondrogenic potential already described for mbMSC in the literature [36,46]. Around 54.1% of the *Alu*-positive mbMSC were also found nearby the neural tube as well as at the ventral root, as shown in Figure 3D–D” and Figure 3E–E” (*n* = 13). We also observed that 33.3% of the mbMSC hybridized with an *Alu* probe (*n* = 8) were found in perineural niches, in association with the peripheral nerves of the embryo (Figure 3F–F”). In Figure 3F”, it is possible to observe the intimate interaction of *Alu*-positive cells with the nerve fibers present in the mesenchyme inferior to the neural tube. Additionally, *Alu*-positive mbMSC (4.1%) were found in a perivascular region, more specifically, in the endothelium of dorsal aorta of embryo at E4.5-5 (HH27) (*n* = 1, Figure 3G–G”), suggesting a possible participation in the processes involving embryonic vasculogenesis.

Moreover, our results showed that mbMSC were able to survive in the chick embryonic microenvironment, as well as being able to integrate into host tissues participating in morphogenesis (including vasculogenesis) together with embryo cells. However, we observed that few cells were found in the embryos in relation to the total number of transplanted cells, since spheroids had hundreds of cells and it was only found a maximum of 15 cells in an embryo.

In contrast to the mbMSC results, bone marrow-derived mesenchymal cells were not found in any of the four engrafted embryos. In this experiment we were able to evaluate, in an unprecedented way, the tropism of mbMSC for perivascular regions contributing to the formation of vessels and in regions proximal to meninges that are extremely instructive for vascular formation in this embryonic phase. These results show the high influence of bmMSC in angiogenesis, suggesting that this capacity could be explored in ischemic clinical therapies.

### 2.4. Endothelial Tube-Like Formation Stimulated by MSC Conditioned Medium

The human umbilical vein endothelial cell (HUVEC) lineage EA.hy926 (ATCC^®^ CRL-2922™), used for endothelial tube formation and migration assay, were evaluated in regard to CD31 expression and functional properties, as shown in Figure S1.

**Figure 4:**
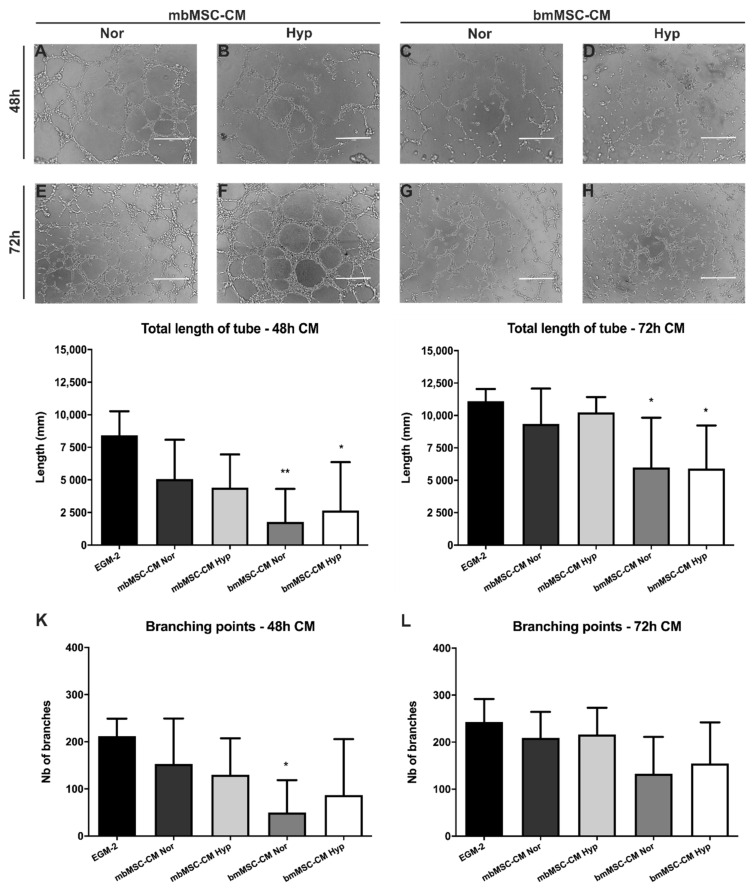
Endothelial tube-like formation stimulated by MSC conditioned medium. Representative phase contrast microscopy images of tubular structures formed by HUVEC EA.Hy926 incubated with (**A**,**B**,**E**,**F**) mbMSC and (**C**,**D**,**G**,**H**) bmMSC conditioned medium derived of (**A**–**D**) 48 and (**E**–**H**) 72 h of culture, carried out in (**A**,**C**,**E**,**G**) normoxia and (**B**,**D**,**F**,**H**) 1% hypoxia, 20 h after plating in Matrigel^TM^ GFR with serum-free MSC conditioned media. (**I**,**J**) Quantification of the total length and (**K**,**L**) branches number of the tubular network formed by HUVEC cultivated with the 48 and 72 h-CM of mbMSC and bmMSC. Values are expressed as mean ± SD (EGM2 48 h, *n* = 4; EGM-2 72 h, *n* = 5; mbMSC and bmMSC-CM, *n* = 5 for each condition); * *p* < 0.05; ** *p* < 0.01 compared to EGM-2. CM, conditioned medium. Nor, normoxia. Hyp, hypoxia. GFR, growth factor reduced. EGM-2, endothelial growth medium 2.

Representative images of tubular structures formed by HUVEC incubated with mbMSC and bmMSC conditioned medium, for 48 and 72 h of culture, carried out in normoxia and 1% hypoxia, are shown in Figure 4A–H.

Initially, the impact of 48 and 72-h conditioned media of mbMSC and bmMSC under normoxic and hypoxic conditions were compared to the angiogenic medium EGM-2. In regard to 48-h conditioned medium, the mbMSC were able to form tubular structures with total length (Figure 4I) and number of branches (Figure 4K) similar to EGM-2 in both O_2_ concentrations. However, only hypoxic bmMSC-CM promoted a similar number of branches compared to EGM-2 (Figure 4K). In regard to 72-h conditioned medium, again all cultured conditions of mbMSC promoted a total length (Figure 4J) and number of branches (Figure 4L) comparable to EGM-2. bmMSC-CM was similar to EGM-2 in relation to the number of branches, in both of O_2_ concentrations (Figure 4L). However, it did not achieve the same efficiency in the total length of the tubes (Figure 4J).

These data suggest that these mediums, mostly, exert an angiogenic effect comparable to EGM-2, which is the commercial medium rich in pro-angiogenic factors. Moreover, CM of longer culture periods, such as 72 h, exhibited higher capacity to form tubular structures for both cells type, showing a longer total length and branch numbers (Figure 4J,L).

Another question to be addressed was whether there was a significant difference between these two cell types conditioned medium when compared to each other. Moreover, the influence of O_2_ concentration on the tubular structure’s formation. In this context, no significant difference was detected between CM derived from both cell types, in different oxygen concentrations, suggesting that the MSC from both sources positively and quite similarly influence the angiogenic capacity of the endothelial cells. Although, we observed a general tendency for mbMSC to be slightly better than bmMSC.

### 2.5. Impact of MSC Conditioned Medium on HUVEC Migration

**Figure 5:**
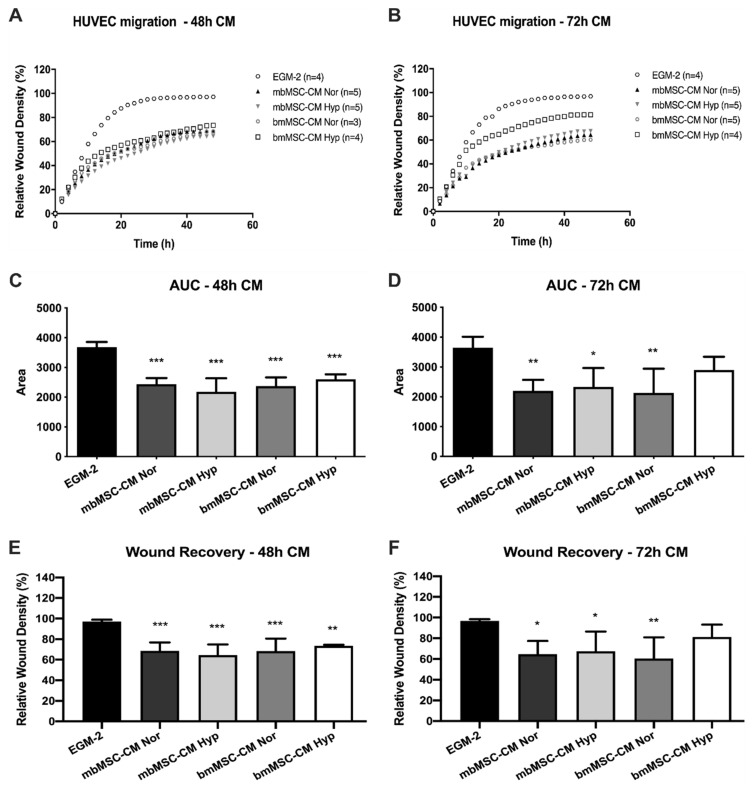
Impact of MSC conditioned medium over HUVEC EA.Hy926 migration by scratch wound assay in vitro. HUVEC migration curves in the presence of serum-free mbMSC and bmMSC-CM, obtained after (**A**) 48 and (**B**) 72-h of culture in normoxia and 1% hypoxia. Sample size for each condition are indicated. (**C**,**D**) Area under the curve (AUC) relative to HUVEC migration curves in the presence of 48 and 72 h-CM of mbMSC and bmMSC. (**E**,**F**) Wound recovery promoted by 48 and 72 h-CM of mbMSC and bmMSC at the point of 48 h after scratch. Values are expressed as mean ± SD; * *p* < 0.05; ** *p* < 0.01; *** *p* < 0.001 compared to EGM-2. CM, conditioned medium. Nor, normoxia. Hyp, hypoxia. EGM-2, endothelial growth medium 2.

HUVEC migration in response to the conditioned media of MSC was evaluated through the scratch wound assay using the relative cell density quantification. Results showed that HUVEC migration curves shift downward when serum-free conditioned medium of mbMSC and bmMSC (Figure 5A,B) were used, independent of culture time, indicating that these medium impacts negatively on migration capacity and possible secreted factors by these cells did not overcome the absence of serum.

Area under the curve (AUC) and wound recovery percentage at the time point of 48 h were also analyzed in order to compare the migratory profile in all conditions. Results showed that mbMSC and bmMSC serum-free conditioned media, obtained after 48 and 72 h of culture, promoted a significant smaller migration of HUVEC when compared to EGM-2, represented by the lowest area under the curve and lowest wound recovery quantification (Figure 5C–F), except for the bmMSC-CM obtained after 72 h of culture in hypoxia (Figure 5D,F). Despite both parameters having differed for only one condition of one cell type, results showed that at the end of the experimental protocol the absence of serum led to less coverage of the scratched area, indicating that fewer cells arrived in the target region compared to EGM-2.

Moreover, we investigate whether conditioned media obtained from normoxic and hypoxic conditions would interfere differently with the migratory capacity of HUVEC and also compare both types of cells with each other. Results showed that there was no difference in HUVEC migration when effects promoted by CM from both cells were compared to each other, showing that CM derived from mbMSC and bmMSC stimulated a similar migratory potential (Figure 5C–F). Although hypoxic bmMSC-CM 72 h was similar to EGM-2 condition, when compared the CM effect of both cells with each other (two-way ANOVA test), we found that hypoxia did not impair or improve the effect of both cells (Figure 5C–F) compared to normoxia.

### 2.6. Angiogenic Related-Factors Expression in MSC

**Figure 6:**
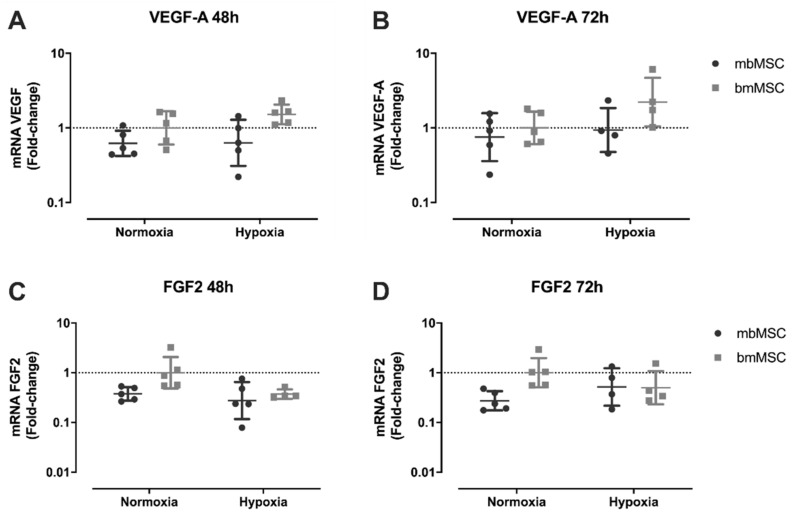
Expression of angiogenic growth factors VEGF-A and FGF2 after 48 and 72 h of culture in a serum-free condition by real time RT-PCR. (**A**–**D**) Comparison of mbMSC and bmMSC expression with each other, at different O_2_ concentrations. (**A**,**B**) VEGF-A expression in MSC cultured for 48 and 72 h, respectively. (**C**,**D**) FGF2 expression in MSC cultured for 48 and 72 h, respectively. For VEGF-A and FGF2 comparison, bmMSC in normoxia was used as a control group. Nor, normoxia; Hyp, hypoxia. RT-PCR, reverse transcription-polymerase chain reaction. VEGF-A, vascular endothelial growth factor A. FGF2, fibroblast growth factor 2.

In order to investigate the mechanism underlying this angiogenic effect promoted by the conditioned media of mesenchymal stromal cells in HUVEC, gene expression of three growth factors important for angiogenesis (PDGF-B, VEGF-A, and FGF2) were evaluated. PDGF-B was expressed only in mbMSC, similarly in all sample conditions, regardless of O_2_ concentration or experimental time (data not shown). Additionally, mbMSC and bmMSC expressed VEGF-A (Figure 6A,B) and FGF2 (Figure 6C,D) after both culture experimental time (48 and 72 h). Moreover, no statistical difference was observed between mbMSC and bmMSC in relation to VEGF-A and FGF2 mRNA levels, regardless of cultivation time (Figure 6A–D).

## 3. Discussion

The present study analyzed the angiogenic properties of human mesenchymal stromal cells derived from bone marrow and menstrual blood in vitro, as well as assessed the MSC migration potential and interaction with the chick embryo tissues in vivo, identifying the location of these cells in a highly instructive and dynamic embryonic microenvironment in an unprecedented way.

In regard to MSC intrinsic angiogenic properties, sprouting assay in 3D culture model was performed as described by Montali et al. [47] in order to evaluate the response of mbMSC and bmMSC spheroids to the initial stages of angiogenesis. The angiogenic process is composed of multiple sequential steps, including tip cell selection, sprout formation, elongation, anastomosis, and vascular stabilization by support cells [7,48,49,50,51]. In this assay, both spheroids exhibited sprouting behavior and primitive tubule network formation. However, mbMSC spheroids showed significantly greater capacity for invasion in the surrounding matrix and formation of multicellular cord-like structures, in relation to bmMSC, in both experimental times evaluated (48 h and 7 days). Montali et al., also showed that bmMSC had a reduced angiogenic capacity when compared to the bone marrow subpopulation called mesangiogenic progenitor cell, evaluated by the in vitro sprouting assay and the in vivo chick embryo chorioallantoic membrane model, where a smaller contribution to neovascular formation was also observed [47]. Moreover, MSC at the invasion sprout front exhibited morphological characteristics similar to endothelial-like tip cells, showing emission of extensions similar to filopodia, and stalk cells organized in a linear manner, contributing to the growth and sprout elongation [52,53,54,55].

MSC spheroids exhibited sprouting behavior and morphology that showed important similarities to in vivo angiogenesis, suggesting that these cells could also be differentiating into an endothelial phenotype. The balance of VEGF-Dll4-Notch signaling is studied as a regulator of the differentiation of tip and stalk fate [56,57,58,59]. EGM-2 medium used in this assay has VEGF in its composition which possibly contributes to this acquired morphology of MSC. Moreover, it has already been demonstrated that MSC angiogenic factors are enhanced with spheroid formation compared to dissociated cells, including the production of VEGF [60,61]. Then, in order to investigate whether these cells also shared phenotypic characteristics with endothelial cells, the presence of the CD31 molecule was evaluated. In order to avoid residual contamination of CD31+ cells due to the heterogeneity of MSC cultures in initial passages, we used cells from passage 5 in this experiment. Like other mesenchymal stromal cells, mbMSC and bmMSC ordinarily did not express CD31 [32,35,37,62,63,64]. However, in this work both MSC appear to be going through endothelial differentiation, since there were positive cells for CD31. Despite few studies in the literature, our data corroborate with other groups that also observed endothelial phenotype in mbMSC and bmMSC [33,65,66], grown under special conditions, as well as are aligned with data described for MSC from other sources, differentiated with specific protocols [67,68,69,70].

In addition to sprouting, the chemotactic migration of cells is an important step in the angiogenic process. So, we sought to mimic an injury microenvironment in vitro through the scratch wound assay and thus evaluate the migration of MSC in response to such stimulus in a 2D system. Our results showed that bmMSC migrated faster than mbMSC in the experimental times of 12 h and 24 h after scratching, as well as had higher AUC. These results suggest that bmMSC can respond more quickly to the trophic factors released by cells in the injury site and, therefore, are promptly mobilized and guided to the region where, probably, has a greater gradient of these factors. However, at the end of the experiment (48 h), cell densities of both MSC are very similar, indicating that they reached the same peak of migration. Jervis et al. reported that bovine bmMSC displayed higher proliferation rates than adipose tissue-MSC but no differences were detected in migration capacity between these cells after 24 h [71].

Additionally, our results were different from those found by Alcayaga and collaborators, who showed a higher migratory ability in mbMSC compared to bmMSC, in the experimental times of 12 and 24 h using the same experiment [72]. These conflicting results might be explained by the SDF-1A/CXCR4 axis which is involved in cells migration to sites of tissue damage and ischemia through hypoxia-inducible factor-1 (HIF-1) [34].

To assess MSC migration potential in vivo and engraftment survival, as well as the interaction of these cells in the embryonic microenvironment, we grafted the MSC-spheroid into the pre-somitic paraxial mesoderm of the embryo, in the region that will give rise to cervical somites at the wing bud level (15th-20th somites) [73]. Cells will receive stimulus from the transplanted region and these instructive signals can influence their migration potential, plasticity and cell destiny, allowing them to adopt different fates in the host organism. Our results showed that only mbMSC were able to survive in the chick embryonic microenvironment, as well as being able to integrate into host tissues, participating in morphogenesis along with embryo cells. mbMSC presented a tropism for presumptive chondrogenic regions of the chick embryo, since 8.3% of these cells were found in territories close to the notochord. Part of the sclerotome cells condenses around the notochord and neural tube, performing endochondral ossification in later stages. Therefore, mesenchymal cells in these regions means that they may also respond to the growth factors involved in the skeletal lineage, such as sonic hedgehog (shh) and noggin, secreted by the notochord and neural tube floor at this embryonic stage [74]. These results corroborated with the potential of this MSC already described in the literature, that when exposed to specific inducing factors, mbMSC can differentiate into cells of chondrogenic and osteogenic lineages [18,33,36,41,46,75].

In addition to the chondrogenic niche, mbMSC have also been found in perineural regions around the neural tube and ventral root. These regions are where the meninges will be formed, which the main functions are to protect the central nervous system and promote blood supply to it. Nimmagadda et al. noted that the first step in developing spinal cord meninges is the expression of the VEGFR-2 receptor in cells derived from the sclerotome immediately adjacent to the lateral surface of the neural tube [76]. This is due to the signals that are released through the neural tube during this developmental phase, including VEGF-A and BMP4, which recruit sclerotome-derived angioblasts and induce the formation of first blood vessels of the perineural vascular plexus [74,77]. Interestingly, 54.1% of mbMSC were found in these regions, suggesting that these cells may be responding to signals secreted by the neural tube at this development stage, which may even be proangiogenic stimuli. In addition, 33.3% of mbMSC were associated with peripheral nerves of the nervous system. Similar results were found by Cordeiro and cols. [78], in which observed that about 90% of the mesenchymal cells derived from adipose tissue (ADSC), transplanted also in the trunk region of the E1.5 (HH8-9) embryo, were found in perineural niches. Mesenchymal cell mobilization and recruitment to perineural regions may also suggest that these cells could participate in such events in vivo in the adult organism, contributing to repair processes of nervous tissue. Differentiation of mesenchymal cells from endometrial origin into neural-like cells has also been tested in vitro by some groups, proving this potential [33,79,80]. However, in vivo differentiation potential of mbMSC in neural-like lineages or another one derived from this somite region (cartilage, bone, muscle, dermis and endothelium) have not been tested in this work, being a possibility for future studies involving these cells.

Lastly, 4.1% of Alu-positive mbMSC migrated to perivascular regions, associating with the dorsal aorta endothelium of the chick embryo E4,5-5 (HH27). This result suggests that mbMSC may participate in events related to embryonic vasculogenesis, contributing to maturation and vascular stabilization, behaving as pericytes, possibly derived from portion of the somatic sclerotome [81]. Perivascular niche of human mesenchymal cells in adult individuals has already been described by several researchers and is currently accepted in the scientific community [82,83,84,85,86,87]. In addition, some authors suggested a possible perivascular localization of mbMSC in the basal and functional layer of human endometrium in vivo, due to the expression of molecules related to pericytes such as CD146, PDGFR- β and SUSD-2, besides mesenchymal markers expression [88,89,90,91]. This reinforces the idea that chick embryonic microenvironment can also be used to check the potential or niche of cells in vivo.

In the second part of the study, we evaluated the paracrine effects of mbMSC and bmMSC’s conditioned mediums on HUVEC EA.hy926 angiogenic capacity. Angiogenic paracrine effects of MSC conditioned medium have already been studied by several groups using different cell sources [72,92,93,94,95,96]. In this work, we sought to evaluate tube-like formation which is the ability to respond to an angiogenic stimulus. Our results showed that the conditioned media of both MSC had a beneficial angiogenic effect on HUVEC, probably due to secretion of angiogenic cytokines in the medium. Although no statistical difference was demonstrated between the two cell sources, we observed a general tendency for mbMSC to be slightly better than bmMSC. Compared to EGM-2, mbMSC had an effect apparently closer to angiogenic medium in regard to tube length and number or branching, than bmMSC. Alcayaga and cols. [72] found that mbMSC promotes higher formation of tubular structures in relation to bmMSC, in both oxygen concentration conditions. However, the authors cultivated MSC with 2% FBS, a condition that was not tested in our study. Besides, Jervis and cols. reported higher tubular networks formation after exposure to concentrated conditioned medium from bovine adipose tissue MSC compared to bmMSC on normoxic condition [71].

Moreover, serum-free conditioned medium of mbMSC and bmMSC, independent of culture time, impact negatively on HUVEC migration capacity compared to EGM-2, represented by the lowest area under the curve and lowest wound recovery quantification, except for the bmMSC-CM obtained after 72 h of culture in hypoxia. In addition, there was no difference in HUVEC migration when CM of both cell lines was compared with each other.

Different conditions (hypoxia and starving) might lead to different protein expression profiles and consequently their effects [97]. Probably this is also one of the reasons we see such a huge difference in the HUVEC’s migration curve with CM-MSC compared to EGM-2. The conditioned medium also has soluble products and metabolites excreted by the cells in culture [98], which in some cases could negatively influence its results. It is important to remember that the EGM-2 condition did not have contact with such products of cellular metabolism.

Since there was, mostly, a benefic angiogenic effect of the conditioned media of MSC on endothelial cells, growth factors expression was evaluated. Both MSC expressed VEGF and FGF2 in both O_2_ concentrations, with no statistical difference between them. Hypoxia frequently can upregulate the expression of some angiogenic cytokines, such as VEGF [99], although this was not observed in this work. Besides that, only mbMSC samples expressed PDGF-B. These data are partially similar to studies developed by Jiang and collaborators that demonstrated that MSC from endometrium expressed significantly higher levels of PDGF mRNA compared to MSC from bone marrow, in normoxia and 1% hypoxia after 24 h of incubation [100]. Similarly, Meng and cols. reported superior capacity of endometrial stem cells in secreting PDGF-BB when compared to umbilical cord-derived mesenchymal cells. In addition, metalloproteases (MMP-3 and MMP-10), GM-CSF and ANG-2 were constitutively present in the culture media of endometrial stem cells. Comparable secretion of angiogenic factors such as VEGF, HGF, and EGF was observed between both cells [33].

These results demonstrate that mbMSC may secrete a distinct set of cytokines and growth factors that could be important therapeutically. Although both MSC have a positive angiogenic effect on HUVEC in vitro, the fact that menstrual blood-derived MSC also express significant amounts of PDGF-B, can suggest a more complete angiogenic contribution, since this cytokine participate both in the formation and in the vascular remodeling of an ischemic tissue.

## 4. Materials and Methods

### 4.1. Isolation and Maintenance of mbMSC and bmMSC

The use of human menstrual blood was approved by the Clementino Fraga Filho University Hospital’s Ethics Committee (52627716.7.0000.5257, 1 February 2016) and the bone marrow was approved by the Bonsucesso Federal Hospital’s Ethics Committee (26046414.7.0000.5253, 1 May 2014). All donors provided signed informed consent in accordance with the principles of the Declaration of Helsinki.

mbMSC were isolated and maintained as previously described by our group [37,62]. Menstrual blood was collected from seven healthy volunteer woman in reproductive age (*n* = 7) using a proper collector cup containing 5 mL of 1× phosphate buffered saline (PBS—LGC BIOTECNOLOGIA, pH 7.2), 100 U/mL penicillin and 100 mg/mL streptomycin (Gibco). Samples were washed twice with PBS and were cultured in high glucose Dulbecco’s modified Eagle’s medium (DMEM; Sigma-Aldrich), supplemented with 20% fetal bovine serum (FBS—Gibco), 2 mM L-glutamine (Sigma-Aldrich), 50 U/mL penicillin and 50 μg/mL streptomycin (Gibco). Medium was changed after two days to remove non adherent cells and cells were maintained at 37 °C in a 5% CO_2_ incubator. For cellular expansion, adherent cells were detached using 0.25% trypsin EDTA (Gibco), counted and sub-cultured.

bmMSC were isolated and maintained as previously described by our group [101]. Briefly, bone marrow aspirates were collected from seven hip surgery patients (*n* = 7; 18 to 60 years old). The material was processed using Ficoll-Paque™ (1.077 g/mL, GE Healthcare) and mononuclear cells were isolated and cultured at the same conditions as mbMSC, except for the serum, which used 15% FBS (Hyclone, Thermo Fisher Scientific, Waltham, MA, US).

All the experiments were performed using these cells at passages P3 to P9. The comparative experiments were performed with the cells in paired passages. Cells were routinely tested for mycoplasma (TaKaRa PCr Mycoplasma detection Kit, TaKaRa Bio Inc, Kusatsu, Shiga, JP; cat. 6601).

### 4.2. 3D Angiogenic Sprouting Assay

Tridimensional spheroids were generated by the hanging drop method. Drops (20 μL) of mbMSC and bmMSC suspensions (1.5 × 10^5^ cells/drop) were laid on the inner surface of a Petri dish lid. To prevent hanging drop dry, 7 mL of PBS were added to Petri dishes and incubated in normoxic condition overnight at 37 °C in 5% CO_2_ for cellular aggregation. Spheroids were gently applied to a Flat bottom 96-well plate (Greiner Bio One, cat. 655986) previously coated with Matrigel^TM^ Growth Factor Reduced (GFR) basement membrane matrix (BD Biosciences, San Jose, CA, US) and cultured in EGM-2 medium (Lonza, cat. CC-3156). Sprouting was evaluated after 48 h and 7 days. Images were acquired by phase-contrast inverted microscope Evos AMG (Thermo Fisher Scientific) and evaluated by measuring the distances between the spheroid edge and the last invading cell of sprouts by Image-Pro Plus software. After image analysis, 3D spheroids were cultured for more than 7 days and then were processed for immunofluorescence.

### 4.3. MSC-Spheroids’ Immunofluorescence

On the 14th day of culture, the sprouting spheroids were fixed in 4% formaldehyde for 30 min at room temperature. Spheroids were permeabilized for 30 min in 0.1% Triton X-100 (Sigma-Aldrich) and blocked in 2% BSA (Sigma-Aldrich, Saint Louis, MO, US), both diluted in PBS, for 2 h at room temperature. All antibodies were diluted in 2% BSA with PBS. For cell washing, 0.05% Tween-20 (Sigma-Aldrich) in PBS was used. CD31 primary antibody (R&D Systems, cat. BBA7, clone 9G11) was diluted 1:50 and incubated with spheroids at 4 °C overnight. The next day, three more washes were performed and then spheroids were incubated with secondary antibody, goat anti-mouse IgG H&L Cy3-conjugated (1:200; Abcam, Cambridge, CAM, UK; cat. ab97035), for one hour at room temperature. After three more washes, spheroids were subsequently stained with Phalloidin antibody conjugated with Alexa Fluor 488 (1:500; cat. A12379, Thermo Fisher) for cytoskeletal labeling. Finally, three washings were performed with PBS, 200 μL of PBS were added per well and 20× magnification photomicrographs were acquired on the LSM 510 laser scanning microscope (Zeiss). The system was linked to the software Zen 2009 that processed the images obtained.

### 4.4. In Vitro Cell Migration Assay

HUVEC, mbMSC and bmMSC migration capacity was evaluated in a scratch assay, where 2 × 10^4^ cells per well were grown separately in 96-well plate (Essen Bioscience Image Lock; Ann Arbor, MI, US) to full confluence. A straight scratch was accurately performed on the cell monolayer using the Wound Maker device (Essen Bioscience, Ann Arbor, MI, US) and cells were washed twice with PBS to remove debris. HUVEC were incubated with mbMSC and bmMSC conditioned medium or endothelial media (EGM-2). However, for mesenchymal stromal cells, after performing the scratch, instead of the conditioned medium, the respective culture medium of each cell type was used. Images at 10× magnification were acquired for each sample every 2 h for a period of 48 h using Incucyte ZOOM automated inverted microscope (Essen Bioscience) to monitor cell migration into the wound area. Migration capacity was quantified using the Relative Wound Density (RWD) algorithm of the software INCUCYTE ZOOM version 2015.A, which allows calculating the density of the wound region as a function of the density of the cell region and is defined by the following Equation (1):

(1) $$\%RWD\left(t\right)=100\times \frac{\left(w\left(t\right)-w\left(0\right)\right)}{\left(c\left(t\right)-w\left(0\right)\right)}$$

where:

w(t) = Density of wound region at time, (t)
c(t) = Density of cell region at time, (t)

### 4.5. mbMSC and bmMSC Spheroids Generation for Chick Embryo Xenograft

Spheroids formed from mbMSC and bmMSC in passage 7 were prepared as described by Brito et al. [102]. Briefly, cells were seeded in high density (5 × 10^5^ cells/mL) and plated in 60 mm Petri dishes, untreated for cell adhesion (J prolab), using the same culture medium previously described, for 48 h.

### 4.6. Chick Embryos Manipulation

The protocols used for handling chick embryos were all approved by the Ethics Committee on the Use of Animals in Scientific Experimentation (Health Sciences Centre of the Federal University of Rio de Janeiro).

Eggs of white Leghorn chicken (Gallus gallus) were obtained from Granja Tolomei (Rio de Janeiro, RJ, BR) and staged according to HH stages [103] or the total number of somites (somite stage, ss). For grafting cells in the somitic region, chick embryos were incubated at 37 °C until they reached at least the thirteenth somite stage (13ss; HH11), on the second embryonic day (E2). On this day, prior to engraftment, albumin excess was aspirated from the eggs by, carefully, opening a small hole in each shells. Then, a window was opened in the upper central part of the egg and India ink (Nankin) was injected into the yolk to allow better visualization of the embryo structures and counting the number of somites. One spheroid of mbMSC or bmMSC (approximately of the size of a chick somite) was grafted into the presomitic mesoderm (PSM) of the presumptive 15–20 somites, at the future wing bud level [73]. The spheroid was inserted into the PSM through a cut in the ectoderm. Grafted embryos (*n* = 8 for each cell type) were reincubated at 37 °C to develop until E5 (HH27). On the fifth day of embryonic development, embryos were euthanized and the specimens prepared for histology.

### 4.7. Genomic In Situ Hybridization with Alu Probes

Embryos were fixed using a Formoy solution [Ethanol (Merck)—37% Formaldehyde (Vetec)—Acetic acid (Merck) in proportions 6: 3: 1, respectively)], dehydrated with ethanol (Merck, Darmstadt, HE, DE) and xylene (Vetec) and embedded in Paraplast (Sigma-Aldrich). Serial sections of 7 μm were performed using a manual microtome (Leica Biosystems, Buffalo Grove, IL, USA) and mounted on silanized slides (Kasvi, São José dos Pinhais, PR, BR).

Human *Alu* probes used in this work were synthesized by PCR from adipose-derived mesenchymal stromal cells as described by Cordeiro et al. [78]. Genomic in situ hybridization of Alu probes on histological sections was performed as previously described [104]. Probes were localized using anti-digoxigenin-AP, Fab fragments (cat. 11093274910, Roche) were diluted 1:2000 in PBS, and were detected using NBT (Roche) and BCIP (Sigma-Aldrich) as substrates. To assemble, slides were washed three times in PBS, counterstained with hematoxylin (Sigma-Aldrich), dehydrated with ethanol/xylene (Merck/Vetec) and mounted with Entellan^®^ new (Merck, Darmstadt, HE, DE). In sections adjacent to those labeled with *Alu* probes, hematoxylin-eosin (Sigma-Aldrich) staining was performed following a routine protocol [105].

The micrographs were taken using the Axioplan microscope with the Axiovision software (Carl Zeiss Microscopy, Oberkochen, BW, DE) and the Pannoramic MIDI II digital slide scanner (3DHISTECH, Budapest, PE, HU) with Caseviewer software (3DHISTECH).

### 4.8. MSC Conditioned Medium Collection

mbMSC and bmMSC were grown in a 60 mm plate at a concentration of 4 × 10^5^ cells for 48 and 72 h with DMEM without serum, in normoxic (21% O_2_) and hypoxic (1% O_2_) conditions. After these times of incubation, the conditioned medium (CM) were collected and centrifuged at 400× *g* for five minutes to remove debris. The supernatants were used for endothelial tube formation and migration assays.

### 4.9. Endothelial Tube Formation Assay

Human umbilical vein endothelial cells at concentration of 2 × 10^4^ cells per well were plated in 96-well plate (Corning^®^, Corning, NY, USA), coated with Matrigel^TM^ GFR basement membrane matrix (BD Biosciences), in the presence of mbMSC and bmMSC conditioned medium, cultured for 48 or 72 h. Endothelial Growth Medium (EGM-2—Lonza, Basel, BS CH) was used as control. After 20 h, images at 10× magnification were acquired by phase-contrast inverted microscope Evos AMG (Thermo Fisher Scientific). Angiogenic capacity of HUVEC was analyzed by quantifying the total length of tubes and the number of branches in each experimental condition using the ImageJ software with angiogenesis analyzer extension.

### 4.10. Gene Expression Analysis of Angiogenic-Related Molecules

Total RNA of mbMSC and bmMSC cultured in normoxic and hypoxic conditions were extracted using RNeasy Mini Plus kit (Qiagen, Germantown, MD, USA). The cDNA was synthesized from 0.5 μg of total RNA by reverse transcription reaction using the High-capacity Reverse Transcription Kit (Applied Biosystems, Carlsbad, CA, USA) following the manufacturer’s instructions. Real-time PCR amplifications were carried out using the 7500 Real-Time PCR System (Applied Biosystems). The amplification reactions were performed in duplicate in a final volume of 15 µL each that contained 3 μL of diluted cDNA (1.5 ng) in RNase-free water, 7.5 μL of 2× power SYBR (Applied Biosystems), 4 μL RNase-free water and 0.5 μL of the primer sense and antisense in the concentration of 10 μM. The amplification conditions were heated up to 50 °C for 2 min, followed by denaturation and activation of Taq polymerase at 95 °C for 10 min, 40 cycles of denaturation at 95 °C for 15 s, annealing at 60 °C for 30 s and extension at 72 °C for 30 s. Transcript levels of the target genes were normalized using GAPDH housekeeping gene as an internal control. Relative gene-specific transcript levels were calculated by the 2^−(ΔΔCt)^ method, where ΔΔCt = [(ΔCt experimental − ΔCt control)]. The ΔCT control represents the ΔCT means of the bmMSC in normoxia. Results were presented on a logarithmic scale at base 10. Primers sequences are shown in Table S1 (Supplementary Materials).

### 4.11. Statistical Analysis

Data was analyzed using Graphpad Prism 8 Software. Student’s *t*-test were used to analyze differences between two groups. One-way or two-way ANOVA followed by Bonferroni multiple comparison post-hoc test was used to analyze differences between three or more groups. Data are presented as mean ± SD, unless stated otherwise. *p*-value less than 0.05 was considered statistically significant.

## 5. Conclusions

In this work, mbMSC demonstrated a greater capacity to invade into the surrounding matrix and to form multicellular cord-like structures in 3D-culture. However, both MSC share endothelial characteristics during angiogenic sprouting, seen by the similarity to tip cells, stalk cells and CD31 expression. Moreover, mbMSC and bmMSC were able to migrate after scratch wound in vitro, nonetheless, only mbMSC demonstrated the ability to migrate and integrate into host tissues in vivo, responding in a particular way to different embryonic signals, being able to engraft in perivascular, perineural and chondrogenic regions.

Conditioned medium of mbMSC and bmMSC were able to stimulate the formation of tubular structures and chemotactic migration in HUVEC regardless of oxygen availability, suggesting that both MSC can contribute efficiently to angiogenesis in several conditions of the microenvironment, including oxygen deprivation. In addition, VEGF-A and FGF2 mRNA were detected in both MSC, besides only mbMSC expressed PDGF-B, suggesting a more complete angiogenic contribution, being able to participate in both formation and vascular remodeling. Finally, we can suggest that mbMSC may prove to be a good alternative for therapeutic angiogenesis, mainly because it is a less invasive source and shows intrinsic and paracrine angiogenic properties in vitro.

## Supplementary Materials

The following are available online at https://www.mdpi.com/1422-0067/21/24/9563/s1, Figure S1: Umbilical cord vein endothelial cell lineage EA.Hy926 maintained morphology, phenotype and functional properties in vitro. Table S1: Primers used in order to evaluate angiogenic-related molecules. Video S1: mbMSC migration on scratch wound assay. Video S2: bmMSC migration on scratch wound assay.

## Abbreviations

ANG-2	Angiopoietin 2
BMP-4	Bone morphogenetic protein 4
EGM-2	Endothelial growth medium
HGF	Hepatocyte growth factor
MSC	Mesenchymal stromal/stem cell
mbMSC	Menstrual blood-derived mesenchymal stromal cells
bmMSC	Bone marrow mesenchymal stromal cells
HUVEC	Human umbilical vein endothelial cells
FGF2	Basic fibroblast growth factor
PDGF-B	Platelet-derived growth factor subunit B
VEGF-A	Vascular endothelial growth factor A
