# Supplementary material for: Intrinsic Angiogenic Potential and Migration Capacity of Human Mesenchymal Stromal Cells Derived from Menstrual Blood and Bone Marrow
Source: Int J Mol Sci. 2020 Dec 15;21(24):9563. doi: 10.3390/ijms21249563 (PMC7765504; doi:10.3390/ijms21249563)
Supplement: Supplementary file 1 [file ijms-21-09563-s001.zip › Suplementary Material/Figure S1 Umbilical cord vein endothelial cell lineage EA.Hy926 maintained morphology phenotype and functional.docx]

*Culture and characterization of Human Umbilical Vein Endothelial Cells (HUVEC) lineage*

The human umbilical vein endothelial cell (HUVEC) lineage EA.hy926 (ATCC® CRL-2922™) was cultured in DMEM high-glucose (4,5 g/L) medium (Sigma-Aldrich) supplemented with 10% FBS (Gibco), 2 mM L-glutamine (Sigma-Aldrich), 50 U/mL penicillin and 50 μg/mL streptomycin (Gibco). These cells were used for endothelial tube formation and migration assays.

For flow cytometry assay, EA.hy926 endothelial cell lineage was detached using trypsin-EDTA (0.25%; Sigma-Aldrich), washed with PBS and centrifuged at 300 × g for 5 minutes. Cells were resuspended in PBS with 0.5% bovine serum albumin (BSA; Sigma-Aldrich) and divided into two tubes with approximately 2 × 10^5^ cells in 100 µL each. Then, 3 µL of monoclonal antibody anti-CD31 conjugated with phycoerythrin (PE) (Biosciences, cat. 555446, clone WM59BD) were added to one tube for 20 minutes, protected from light, at 4°C, according to the manufacturer's instructions. Unstained cells were used as an auto fluorescence control. In both tubes, the cell viability marker 7-amino-actinomycin D (7-AAD) (BD Biosciences, cat. 559925) was used in order to select viable cells for analyses. After the staining period, cells were washed with PBS and centrifuged at 300 × g for 5 minutes. Cells were acquired using Accuri C6 flow cytometer (BD Biosciences) and CD31 expression was analyzed with FlowJo software version .10.1.

For immunofluorescence assay, HUVEC were plated (2 × 10^4^ cells/well) over a flat bottom 96-well plate (Greiner Bio One, cat. 655986) coated with Matrigel^TM^ GFR basement membrane matrix (BD Biosciences) and maintained by 24 hours in EGM-2 medium (Lonza). After this period, cells were washed with PBS and fixed with 4% paraformaldehyde solution for 20 minutes at room temperature. Then, fixed cells were washed three times with PBS and permeabilized with 0.1% TRITON X-100 for 30 minutes. Unspecific binding sites were blocked with 2% BSA for one hour. CD31 primary antibody (R&D Biosystems, cat. BBA7, clone 9G11) at 1:50 dilution in 2% BSA with PBS solution were added to the fixed cells and incubated overnight at 4°C. In the following day, wells were washed three times with 0.05% Tween-20 for five minutes each and then incubated with the secondary antibody conjugated to Cy3 (Abcam, cat. ab97035) also diluted in a PBS solution containing 2% BSA, for one hour at room temperature. After, three more washes were performed and Phalloidin antibody conjugated with Alexa Fluor 488 (Thermo Fisher, cat. A12379) was used to show the cytoskeleton. Finally, a series of three washes with PBS was performed again and photomicrographs were acquired using LSM 510 (Zeiss) microscope and processed with Zen 2009 software.

Therefore, HUVEC EA.Hy926 lineage cultured in basal medium, directly plated over a plastic-treated surface, showed a homogeneous flattened morphology, which is characteristic of a mature endothelial cell, called cobblestone as shown in Figure S1A.

In order to assess whether the immunophenotypic characteristics and functionality of this HUVEC still remained unchanged, even after a long time of cultivation and through several passages, the presence of CD31 surface molecule was analyzed. Flow cytometry revealed that about 79.9% of viable cells expressed CD31 on the membrane surface (Figure S1B and S1C). Moreover, these HUVEC presented functional characteristics of endothelial cells once they were able to form tubular structures in Matrigel^TM^ GFR coated plate, in the presence of EGM-2 culture medium (Figure S1D). Immunofluorescence assay performed after tubular structure formation, confirmed the presence of CD31 molecule and phalloidin allowed cytoskeleton visualization (Figure S1E – G).


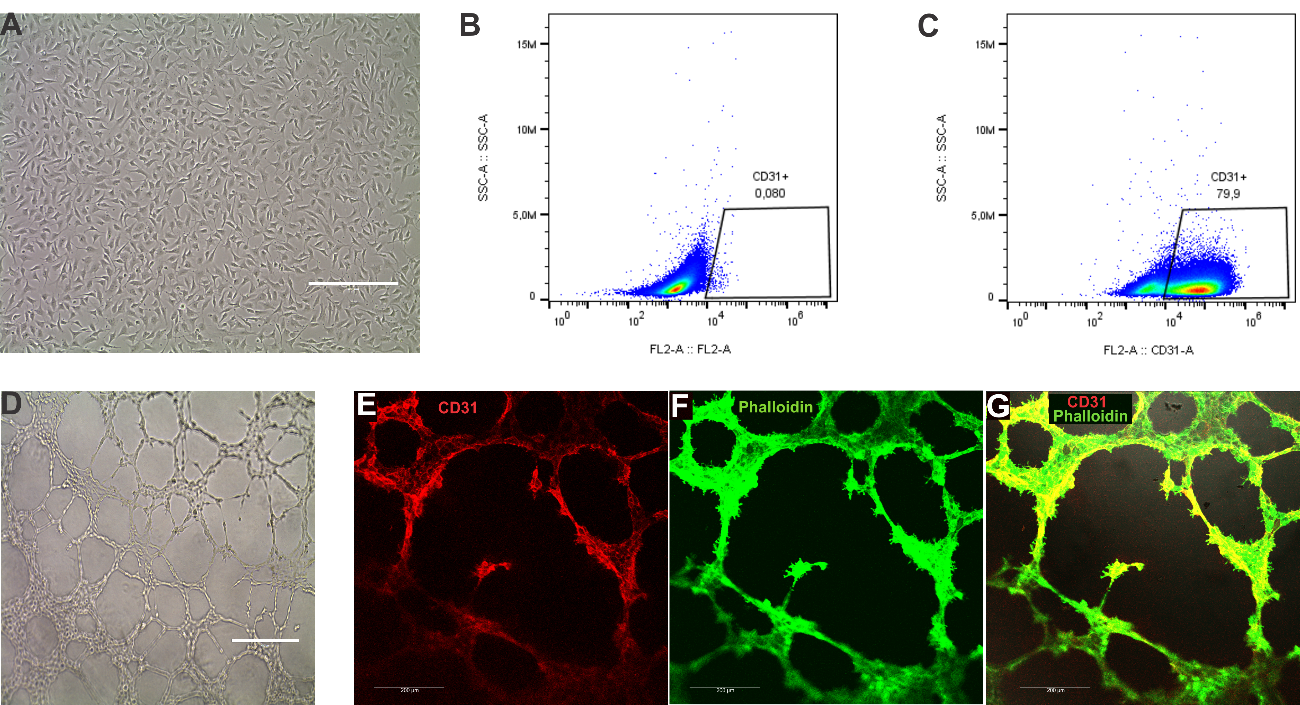


**Figure S1:** Umbilical cord vein endothelial cell lineage EA.Hy926 maintained morphology, phenotype and functional properties in vitro. (A) Phase contrast microscopy image of HUVEC EA.Hy926 showed a homogeneous characteristic cobblestone morphology in 2D culture. (B and C) Representative dot plots of HUVEC EA.Hy926 lineage phenotypic profile by flow cytometry. (B) Unstained HUVEC were used for fluorescence control. (C) Viable cells were mostly positive for CD31 surface molecule (79.9%). (D) Phase contrast microscopy of tubular structures network formed by HUVEC EA.Hy926, 20 hours after plating in Matrigel^TM^ GFR with EGM-2 medium, showed extensive tubular network formation. (E-G) Photomicrographs of HUVEC EA.Hy926 immunofluorescence. (E) Endothelial marker CD31 is shown in red. (F) Cytoskeleton was evidenciated by filamentous actin phalloidin antibody as shown in green. (G) Merged image showing HUVEC cytoskeleton (green) and CD31 (red). All scale bars are indicated in each image.
