# Supplementary figures and images for: Intrinsic Angiogenic Potential and Migration Capacity of Human Mesenchymal Stromal Cells Derived from Menstrual Blood and Bone Marrow
Source: Int J Mol Sci. 2020 Dec 15;21(24):9563. doi: 10.3390/ijms21249563 (PMC7765504; doi:10.3390/ijms21249563)

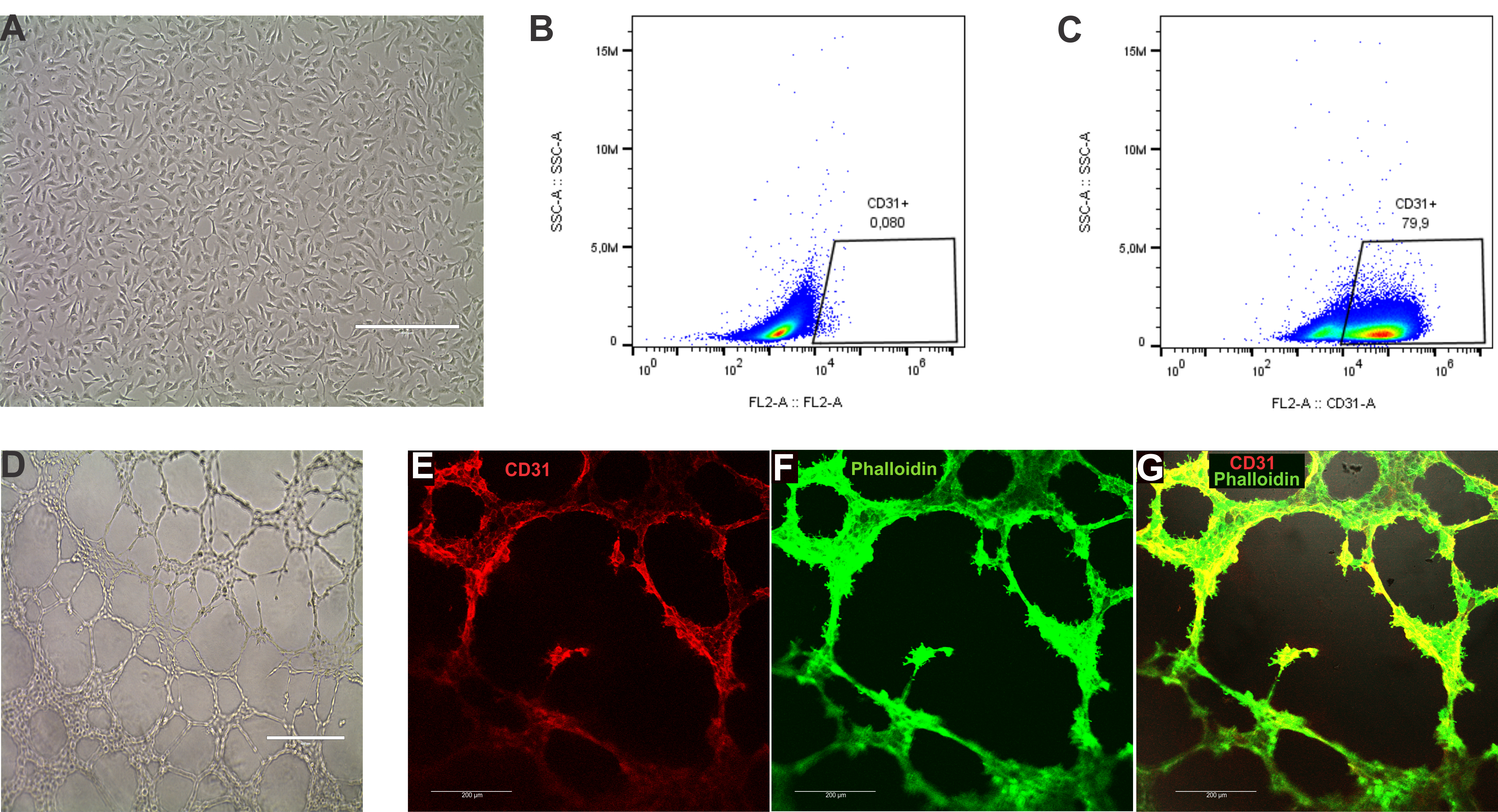

Supplement: Supplementary file 1 [file ijms-21-09563-s001.zip › Suplementary Material/Figure S1.tif]
