# Supplementary material for: Intrinsic Angiogenic Potential and Migration Capacity of Human Mesenchymal Stromal Cells Derived from Menstrual Blood and Bone Marrow
Source: Int J Mol Sci. 2020 Dec 15;21(24):9563. doi: 10.3390/ijms21249563 (PMC7765504; doi:10.3390/ijms21249563)
Supplement: Supplementary file 1 [file ijms-21-09563-s001.zip › Suplementary Material/Table S1 Primers used in order to evaluate angiogenic-related molecules..doc]

| **Gene** | **Forward sequence**  **Reverse sequence** | **Product size (bp)** |
| --- | --- | --- |
| VEGF-A | AGGGCAGAATCATCACGAAG  GGATGGCTTGAAGATGTACTC | **130** |
| FGF2 | ACCCTCACATCAAGCTACAAC  AAAAGAAACACTCATCCGTAACAC | **141** |
| PDGF-B | ATGATCTCCAACGCCTGC  TCAGCAATGGTCAGGGAAC | **145** |
| GAPDH | ACCATGGGGAAGGTGAAGGT CATGGGTGGAATCATATTGG | **163** |

Table S1: Primers used in order to evaluate angiogenic-related molecules.
